# Identifying Evidence-Based Strategies in a Digital Mental Health Intervention for Depression: Qualitative Content Analysis

**DOI:** 10.2196/84030

**Published:** 2026-04-16

**Authors:** Geneva K Jonathan, Jenna Y Sung, Heyli T Arcese, Phoebe Holz, Kathryn H Bentley, Emily E Bernstein, Hilary M Weingarden, Jennifer L Greenberg, Oliver T Harrison, Sabine Wilhelm

**Affiliations:** 1 Department of Psychiatry, Massachusetts General Hospital, 185 Cambridge Street, 2nd Floor, Boston, MA, 02114, United States; 2 Department of Psychiatry, Harvard Medical School, Boston, MA, United States; 3 Koa Health, London, United Kingdom

**Keywords:** digital mental health, cognitive behavioral therapy, behavior change techniques, depression, content analysis, treatment fidelity, therapist-guided, smartphone intervention, transparency, qualitative

## Abstract

**Background:**

Depression is one of the leading causes of disability worldwide. Cognitive behavioral therapy (CBT) is an effective treatment, but it is difficult to access due to clinician shortages, waitlists, and logistical barriers. Smartphone-based CBT interventions offer a scalable alternative to traditional face-to-face care, but few provide transparency regarding how closely they adhere to evidence-based therapeutic principles. Understanding what therapeutic components are included in interventions helps clinicians and patients determine whether they follow CBT principles and how they might help reduce depressive symptoms.

**Objective:**

This study aimed to characterize the therapeutic content of Mindset (Koa Health), a therapist-guided smartphone intervention for depression, by identifying the core CBT techniques it delivers and the specific behavioral strategies the app uses to put those techniques into practice.

**Methods:**

A qualitative content analysis was conducted on all 393 unique intervention pages of Mindset. Using established CBT strategy definitions and the behavior change technique (BCT) Taxonomy version 1 (BCTTv1), coders independently evaluated each page using a collaborative consensus approach. Interrater agreement was 93.75% for CBT and 93.62% for BCT coding. Descriptive statistics (frequency, mean, and SD) and overlap between the two were calculated.

**Results:**

All 16 core CBT techniques were identified. CBT techniques were used a total of 528 times (mean per module 66.0, SD 56.0). The most frequently used techniques included psychoeducation (164/325, 50.5% of pages), skill building (110/325, 33.8%), cognitive restructuring (46/325, 14.2%), activity scheduling (42/325, 12.9%), and self-monitoring (39/325, 12%). Across modules, 37 of 93 possible BCTs were coded 878 times (mean per module 109.8, SD 92.0) across 13 of 16 BCTTv1 categories. The most frequently applied BCT categories were shaping knowledge (205/325, 63.1% of pages), repetition and substitution (138/325, 42.5%), and feedback and monitoring (113/325, 34.8%). Overlap between the 2 frameworks was common, with the most frequent CBT-BCT pairings being psychoeducation (CBT technique)×Shaping knowledge (BCT category; appearing together on 119 pages), skill building×Shaping knowledge (80 pages), activity scheduling×Shaping knowledge (42 pages), and activity scheduling×Repetition and substitution (42 pages).

**Conclusions:**

Mindset demonstrates coverage of CBT techniques and alignment with evidence-based BCTs. This study is the first to introduce mechanism mapping, a dual-coding approach that describes the presence of therapeutic strategies and how they are behaviorally operationalized, addressing a gap in digital mental health transparency. Unlike existing content evaluations that use presence or absence checklists, our framework captures implementation depth through systematic documentation of behavioral scaffolding. This replicable methodology enables researchers to evaluate therapeutic fidelity, supports clinicians in making evidence-informed recommendations for digital mental health treatments, and provides a foundation for the development of adaptive interventions that can enhance real-world treatment outcomes for individuals with depression.

## Introduction

Depression is a severe and common illness that affects more than 300 million people worldwide, making it one of the leading causes of disability [1,2]. Its prevalence has risen in recent years [3], increasing the disease’s burden on individuals and health care systems [4]. Depression impairs quality of life [5], disrupts cognitive functioning [6-8], and increases suicidal thoughts and behaviors [4,9]. Cognitive behavioral therapy (CBT) is among the most effective treatments for depression, with extensive evidence supporting its ability to reduce symptom severity [10-12] and improve functioning [13] and quality of life [14]. However, access to traditional face-to-face CBT is limited due to clinician shortages, long waitlists, geographic limitations, out-of-pocket costs, stigma, and motivational difficulties inherent to depression [15-19].

Digital interventions have emerged to address barriers to traditional face-to-face care. Multiple meta-analyses of app and web-based interventions for individuals with moderate-to-severe depression demonstrate moderate reductions in symptom severity relative to usual care or minimal control conditions, with pooled effect sizes falling in the moderate range (standardized mean difference 0.50‐0.62) [20-22]. However, these average effects provide limited insight into what users are actually receiving in digital “CBT-based” interventions. Systematic reviews of these apps consistently show that adherence to evidence-based CBT or behavioral activation protocols is as low as 15%, and that commercially available apps do not fully meet established guideline, protocol, or treatment manual standards [23-29].

The gap between interventions labeled as “CBT-based” and their actual implementation of CBT becomes apparent when examining app content in detail. Content analyses of existing depression apps show that many include only a small number of evidence-based treatment elements (median 3 per app), with some of the most “hallmark” CBT strategies, such as cognitive restructuring, problem-solving, and relapse prevention, absent or underrepresented [25]. Even when CBT components are present, they may be implemented superficially, with limited tailoring and follow-up. For example, a review of 28 unguided depression apps and web programs found that even though behavioral activation was “present” in many programs, the strategy was rarely broken into achievable steps, anticipated barriers were not addressed, and few programs checked whether planned activities were completed, despite behavioral activation being one of the most commonly included components in CBT for depression [27]. These findings suggest that existing methods for evaluating the presence and depth of therapeutic strategies fall short of capturing the concrete implementation features that determine whether a therapeutic component functions as structured skills training (likely to achieve a greater therapeutic effect) or as a brief, one-off exercise.

Despite this limitation, existing reviews and meta-analyses have not expanded their approach to characterizing how CBT is operationalized within these tools. Most syntheses classify interventions using broad labels such as “CBT-based” or by the presence or absence of high-level components, which are often derived from intervention descriptions, stated theoretical orientations, or protocol-level summaries rather than a systematic review of the delivered in-app content and exercises themselves [21,29,30]. As a result, interventions that differ meaningfully in their therapeutic design are often treated as equivalent, obscuring variation in the active ingredients users are exposed to. Even content-focused evaluations typically summarize interventions at the program or app level, focusing on content frequency or presence and providing little information about the distribution, sequencing, or repetition of specific therapeutic techniques [24,25]. Without more granular and methodological characterization of therapeutic content, it is difficult to link intervention design to engagement, behavior change, or clinical outcomes, or to identify which components of digital CBT are most essential for effective depression treatment. However, detailed characterization of therapeutic content only addresses part of the challenge. Knowing what strategies are present does not explain how they produce change.

The mechanisms through which digital interventions exert their effects are poorly understood [22,30]. In traditional face-to-face CBT, symptom improvement is thought to arise from the coordinated enactment of cognitive, behavioral, and self-regulatory processes supported through structured practice, homework, and therapist feedback [31]. However, it is unclear whether digital CBT interventions engage these same change processes, particularly given their asynchronous format, reduced clinician involvement, and reliance on self-directed engagement. A critical but underexplored question is how digital interventions operationalize these strategies; that is, through what behavioral mechanisms do they prompt users to engage in the cognitive and behavioral work that facilitates change. Without understanding how digital interventions translate therapeutic strategies into concrete, repeated user behaviors, it is difficult to determine whether they provide structured practice opportunities that established CBT theory suggests are necessary for skill acquisition and symptom change [32,33].

To understand how digital CBT engages change mechanisms, we must recognize that CBT strategies are behavioral in nature [31]. Cognitive restructuring involves identifying and examining thoughts, activity scheduling requires planning and completing activities, and behavioral experiments entail designing and implementing behavioral tests. Critically, the same strategy can be delivered through very different user tasks, such as written thought record versus brief reflection prompts, or a full weekly activity plan versus a single daily goal. Because CBT strategies can be enacted in multiple ways, we need to move beyond describing whether they are mentioned to specifying how strategies are translated into user actions and practice opportunities.

Behavior change techniques (BCTs) provide a structured way to characterize how interventions translate therapeutic strategies into user actions that support skill enactment and change. BCTs are the smallest identifiable “active ingredients” designed to change behavior, for example, prompting self-monitoring, providing instruction, or encouraging repetition [34]. The BCT Taxonomy version 1 (BCTTv1) has been widely applied across digital behavioral domains, including medication adherence [35], physical activity [36,37], alcohol [38], smoking cessation [39], and condom use [40]. However, the application of BCT in digital mental health is comparatively limited [41-43]. Pairing CBT strategies with BCT coding enables a systematic approach to understanding how therapeutic components are behaviorally operationalized, for example, how activity scheduling is supported through action planning, self-monitoring, or behavioral prompts. This aligns with growing calls to identify active ingredients and clarify the processes through which digital interventions achieve effects [44,45].

This paper evaluates Mindset (Koa Health), a therapist-guided smartphone CBT intervention for depression, to advance transparency and mechanistic understanding in digital mental health. Mindset was selected because it has demonstrated clinical outcomes in an open trial [46] and uses a therapist-guided delivery model associated with superior effectiveness compared to self-guided approaches [47,48]. Most importantly, we had complete access to all intervention content for comprehensive coding. In the 8-week trial of Mindset (n=28), participants showed significant reductions in clinician-rated depression severity on the Hamilton Depression Rating Scale [49] from baseline (mean 19.1, SD 5.0) to posttreatment (mean 10.8, SD 6.1), with a large effect size (Hedges g=1.47; *P*<.001; mean reduction 7.8 points, 95% CI 5.2‐10.5), with gains maintained at 3-month follow-up [46]. Large effects were also observed for self-reported depression, functioning, and quality of life [46].

To better understand how Mindset supports change and delivers its clinical effects, we aimed to identify and describe the CBT strategies and BCTs present in Mindset and explore how BCTs operationalize CBT strategies by describing their overlap. Our overarching goal is to propose a methodological framework for describing therapeutic content that can be replicated to support digital mental health transparency.

## Methods

### Study Design

We conducted a qualitative content analysis of all intervention pages (ie, all app content) in Mindset, a therapist-guided smartphone CBT intervention for depression.

### Ethical Considerations

The original open trial of Mindset for Depression took place at Massachusetts General Hospital in Boston, Massachusetts, between May 2022 and February 2023. It was approved by the Institutional Review Board of Massachusetts General Hospital (protocol 2020P001958) and was registered on ClinicalTrials.gov (NCT05386329). All trial participants provided informed consent before the initiation of study procedures and were given the option to withdraw at any time. Participants in the original trial were compensated US $25 at each of 3 assessment time points (midtreatment, posttreatment, and 3-month follow-up). Given that it was a content analysis of the intervention materials only, this study did not include any participant data, user-generated content, or personally identifiable information. Therefore, no additional consent was collected. No images, screenshots, or other materials in this paper or supplementary files contain any participant data or identifiable information; all content reflects only the intervention’s standardized therapeutic materials. The full open trial, including a detailed description of study methods and results, is reported elsewhere [46].

### Mindset Intervention Delivery

Mindset is a therapist-guided smartphone CBT intervention for depression consisting of 8 modules delivered across 8 weeks [46]. The intervention is grounded in CBT theory, which posits that improvements in depression are facilitated by changes in thoughts, behaviors, and emotional responses [50].

Participants had immediate access to the intervention after completing baseline assessments. They were instructed to use the app independently while attending weekly 15‐ to 20-minute therapist sessions conducted via videoconferencing (Health Insurance Portability and Accountability Act [HIPAA]-compliant) to review progress and receive support. Therapists were licensed clinical psychologists trained in CBT for depression, and, because of licensure requirements, participants were required to reside in Massachusetts.

The program began with a brief onboarding sequence that introduced CBT principles and app navigation (Table 1). Modules followed a typical CBT progression, beginning with psychoeducation and self-monitoring, moving into behavioral activation, and ending with cognitive restructuring and relapse prevention [50]. Module 1 could be completed at the participant’s own pace, whereas Modules 2‐8 required a minimum of 7 days per module to support practice and skill consolidation. During Module 2, participants were prompted to self-monitor by logging at least 3 activities they engaged in and the corresponding moods each day. From Module 3 onward, participants implemented behavioral activation by scheduling at least 3 activities per week aligned with 7 value domains (health and wellness, causes and community, spirituality, creative pursuits, career and education, personal relationships, and day-to-day tasks), then logging mood after each activity. The activity library included 102 options, and activity scheduling continued through Modules 4‐8. Although therapeutic content was standardized, participants’ activity choices and the extent to which they revisited the activity scheduling and logging pages allowed for optional individual tailoring or personalization.

**Table 1.** Mindset intervention overview, module-by-module.

Module	Description	Number of pages		
Unique	Repeated	Total
Onboarding	Introduction to intervention and how to use	10	0	10
Module 1: Exploring How We Think	Cognitive restructuring to challenge unhelpful thinking	135	0	135
Module 2: Activities and Mood	Self-monitoring of activities and associated moods	28	0	28
Module 3: Goals and Scheduling	Identify personal values, set goals, and schedule activities aligned with values	24	7	31
Module 4: Establishing a Rhythm	Mindful breathing, continued scheduling of activities	19	7	26
Module 5: Keeping Momentum	Mindful grounding, continued scheduling of activities	9	7	16
Module 6: Planning Around Priorities	Skills to let go of unhelpful thoughts, continued scheduling of activities	9	7	16
Module 7: Examining Core Beliefs	Modification of core beliefs and building self-esteem, continued scheduling of activities	26	7	33
Module 8: Preparing for the Future	Relapse prevention, reflect on progress and learned skills, continued scheduling of activities	23	7	30
Total		283	—^a^	325

a Not applicable.

All data and therapeutic materials were sourced directly from the Mindset app, which included 393 unique app pages across onboarding content and modules (Table 1), totaling 943 pages when repeated content was included. No materials or pages from the app were excluded from analysis. Content was accessible only within the smartphone app and was not available as external files or in print. On average, there were 39.3 pages per module (SD 44.8). Content was delivered in the app via text, though some pages, such as the introductory module, mindfulness and grounding exercises, included the option to watch the content via video.

### Coding Framework

We used a dual-coding approach to systematically identify what therapeutic strategies were delivered and how. This approach, which we call mechanism mapping, pairs established CBT technique definitions [31,50] with the BCTTv1 [34] to describe the therapeutic content included and its behavioral operationalization.

### CBT Techniques

We first identified and labeled CBT techniques present in Mindset to determine which therapeutic strategies were included and to evaluate alignment with established CBT protocols for depression (Table S1 in Multimedia Appendix 1). The CBT coding framework consisted of 16 techniques found in manualized CBT protocols for depression [25,31,50]. Refer to Table S1 in Multimedia Appendix 1 for coding definitions.

### BCTs

We applied the BCTTv1, a validated framework comprising 93 techniques organized into 16 hierarchically clustered categories [34,51,52]. The first author (GKJ) completed training through the official online platform [51]. The 16 categories and their definitions are fully described by Michie et al [34] and in Table S2 in Multimedia Appendix 1.

We chose BCTTv1 because of its ability to characterize the “ingredients” that operationalize change in a digital CBT intervention, consistent with a mechanisms-focused approach [34]. It also provides a validated, fine-grained taxonomy for identifying discrete, observable techniques embedded in app-based content, that is, what users see and do on each screen, making it particularly appropriate for therapy apps that translate evidence-based strategies into modular, self-guided activities. Other frameworks, such as the Theoretical Domains Framework and the Capability, Opportunity, Motivation–Behavior (COM-B) theory, describe why behavior changes by organizing determinants, but do not enumerate concrete techniques [53,54]. Although BCTTv1 was originally developed for health behavior interventions, its structure aligns well with CBT’s emphasis on observable behavioral and cognitive skills [50].

### Coding Procedures

The raw text of the Mindset app was organized page-by-page to preserve the intervention’s structure during coding. A collaborative coding approach was used to identify CBT techniques and BCTs [55]. Techniques were coded based on the presence of explicit content, exercises, or instructions; implied techniques were not coded.

### Coder Roles, Independence, and Workflow

Coding was conducted by 2 doctoral-level clinical psychologists (GKJ and JYS) and 1 bachelor’s-level research assistant (HTA), all familiar with CBT for depression. None were involved in the initial development or testing of the Mindset intervention. The first author (GKJ) independently coded each intervention page in Dedoose (SocioCultural Research Consultants, LLC) [56]. Two additional coders (JYS and HTA) reviewed the codes and documented disagreements in memos. Discrepancies were discussed collaboratively using the memos as well as CBT and BCTTv1 definitions until consensus was reached.

### Interrater Agreement

To assess coder agreement, we report percentage agreement as the reliability metric [57]. Percentage agreement is appropriate for qualitative content analysis with multiple potential codes (eg, different pages of an intervention included multiple techniques or skills), because kappa statistics can be overly conservative when the number of possible categories is large relative to the sample size [57,58]. Before consensus, agreement was 93.62% for BCT codes (822/878) and 93.75% for CBT codes (494/528).

### Analysis

We calculated the frequency and distribution of CBT techniques and BCT categories across Mindset, including mean applications, SD, range, and percentage of total app pages.

For ease of interpreting results, we summarize BCT categories rather than each technique, and we prioritize CBT techniques and BCT categories that contribute ≥10% of total codes for reporting.

To prevent repeated content from inflating counts, we weighted pages that participants were guided to revisit multiple times. For example, activity scheduling pages appeared in Modules 3‐8, with instructions to schedule at least 3 activities per week. We applied a weighting factor equal to the average number of activities scheduled per participant divided by the total number of possible activity pages (n=110). This weighting assumption was based on participant instructions provided in the app. After applying this weighting factor, the 110 activity library pages in each of Modules 3-8 were reduced to 7 weighted pages per module, yielding an analytic dataset of 325 pages (Table 1) used as the denominator for all frequency and percentage calculations.

Data aggregation and analysis were conducted using R version 4.4.2 (R Core Team) [59]; a heatmap was developed using the *pheatmap* package [60], and figures were finalized in Canva (Canva Pty Ltd) [61]. Multimedia Appendix 1 includes detailed documentation of coding distributions.

### Reporting Standards

This study follows the TIDieR (Template for Intervention Description and Replication) reporting guideline to promote transparency and replicability in describing intervention content and delivery [62]. A completed TIDieR checklist is provided in Checklist 1.

## Results

### CBT Techniques

**Figure 1.**
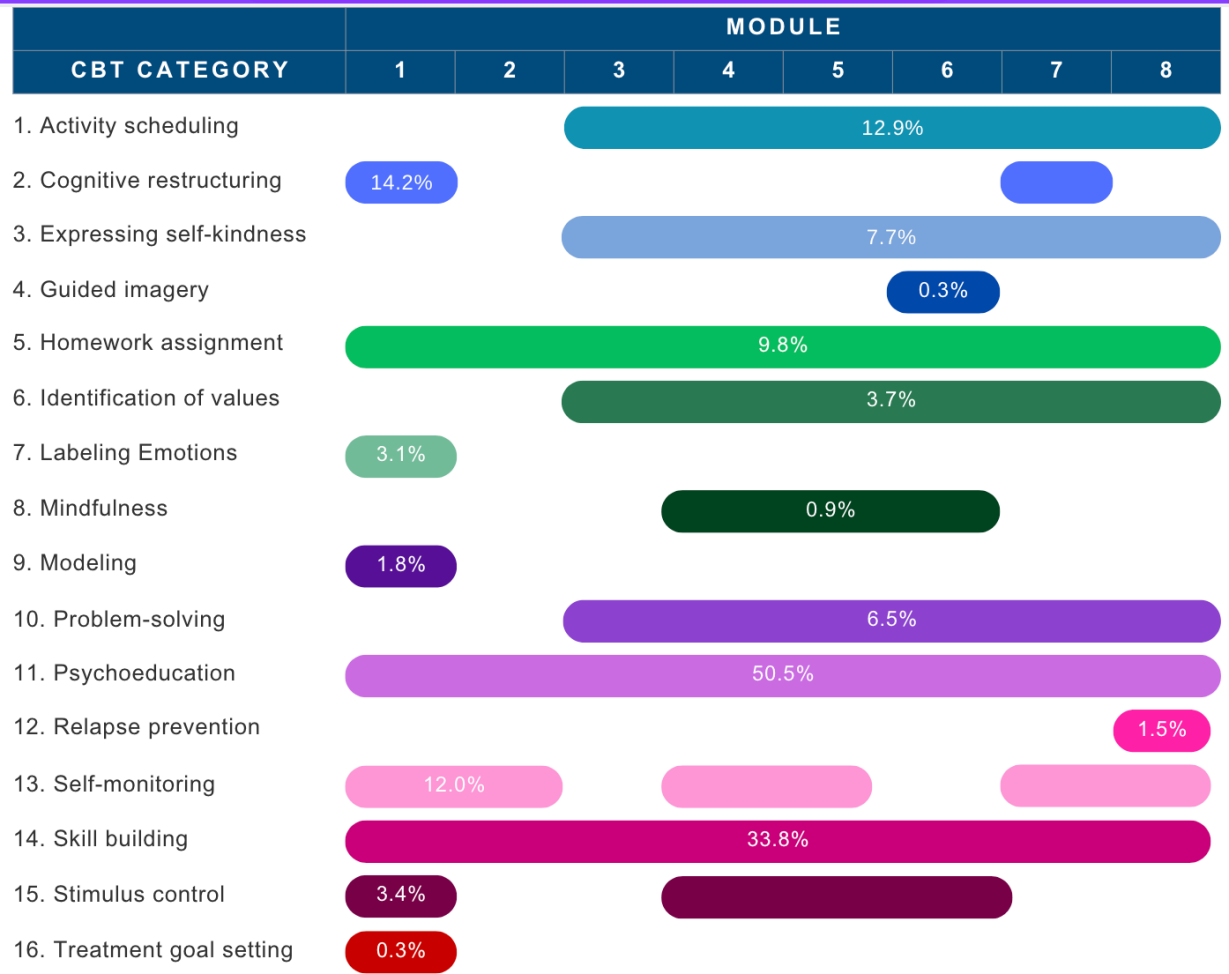
Distribution of cognitive behavioral therapy techniques across intervention modules in the therapist-guided Mindset smartphone program for depression. This figure summarizes when each of the 16 cognitive behavioral therapy techniques was delivered throughout the 8 modules. Horizontal bars represent the percentage of app pages (N=325) within each module that included the cognitive behavioral therapy strategy. CBT: cognitive behavioral therapy.

All 16 CBT techniques were represented and applied 528 times (mean 66.0 per module, SD 56.0) throughout the intervention. Figure 1 shows the distribution of CBT techniques by module, and Table 2 reports the descriptive summary of identified strategies. Full frequencies and distributions are reported in Multimedia Appendix 1.

**Table 2.** Cognitive behavioral therapy techniques in the Mindset smartphone intervention for depression.^a^

Rank	CBT^b^ category	Top module	Pages, n (%)	Mean per module when present (SD)	Examples of implementation
1	Psychoeducation	1	164 (50.5)	20.5 (23.1)	Explanation of the difference between sadness and depression
2	Skill building	1	110 (33.8)	13.8 (12.9)	Prompting participants to log 3 activities a day
3	Cognitive restructuring	1	46 (14.2)	23.0 (21.2)	Identifying a negative thought or belief and typing in a more balanced thought
4	Activity scheduling	3-8	42 (12.9)	7.0 (0.0)	Scheduling 3 activities per day and rating the mood for each activity
5	Self-monitoring	1	39 (12.0)	5.6 (13.8)	Logging 3 activities per day and rating mood for each activity
6	Homework assignment	1	32 (9.8)	4.0 (0.5)	Direction to at least 3 log activities in order to move to next module
7	Expressing self-kindness	1	25 (7.7)	4.2 (2.4)	“Write an encouraging note to self”
8	Problem-solving	1	21 (6.5)	2.6 (0.8)	Ask participants to identify and write down barriers to planned activities in the app
9	Identification of values	1	12 (3.7)	1.5 (1.9)	Selection of activities aligned with personal values
10	Stimulus control	1	11 (3.4)	2.8 (3.5)	“Turn off notifications while practicing mindfulness”
11	Labeling emotions	1	10 (3.1)	10 (0.0)	Encouragement to reflect on and name what emotion they feel after a challenging situation
12	Modeling	1	6 (1.8)	6.0 (0.0)	Demonstration of how to set a SMART^c^ goal
13	Relapse prevention	8	5 (1.5)	5.0 (0.0)	Preparing a plan for anticipated stressful events
14	Mindfulness	4-6	3 (0.9)	1.0 (0.0)	Guided breathing exercise with attention to present-moment sensations
15	Guided imagery	6	1 (0.3)	1.0 (0.0)	“Visualize placing each thought on a cloud and watching it drift away”
16	Treatment goal setting	1	1(0.3)	0.1 (0.4)	Set goal in alignment with values

a Cognitive behavioral therapy strategies were coded 528 times throughout all 8 intervention modules. As such, percentages are reported as a percentage of the total app pages (N=325).

b CBT: cognitive behavioral therapy.

c SMART: specific, measurable, achievable, relevant, and time-bound.

### High-Frequency Techniques (≥10%)

Psychoeducation was present in all modules and accounted for more than half of the intervention pages (164/325, 50.5%), followed by skill building (110/325, 33.8%) and cognitive restructuring (46/325, 14.2%), both of which were most present in Module 1. Activity scheduling (42/325, 12.9%) appeared consistently across Modules 3‐8, in alignment with repeated practice of behavioral activation skills. Self-monitoring (39/325, 12.0%) was present intermittently throughout the intervention, but most focused on modules that asked users to log and observe mood changes linked to activities.

### Low-Frequency Techniques (<10%)

Homework assignments, self-kindness, problem-solving, and value identification appeared on 3%‐10% of the pages. Labeling emotions, mindfulness, guided imagery, and relapse prevention were less frequent (<5%).

### BCTs

**Figure 2.**
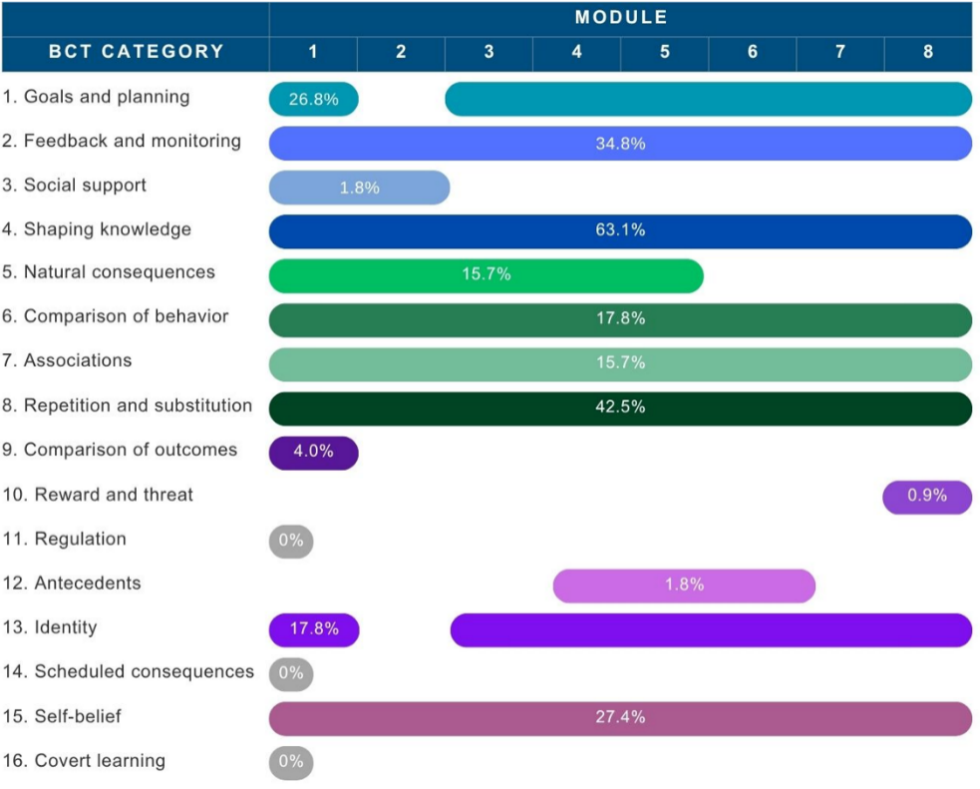
Distribution of behavior change technique categories across the therapist-guided Mindset smartphone cognitive behavioral therapy intervention for depression. Each of the 13 behavior change technique categories identified in Mindset was delivered throughout the 8 modules. Horizontal bars represent the percentage of app pages (N=325) within each module that included the cognitive behavioral therapy strategy. BCT: behavior change technique.

The intervention included 37 BCTs (39.8% of the taxonomy), from 13 of 16 categories, applied 878 times (mean 109.8, SD 92.0) across modules. Figure 2 shows the distribution of BCTs by module, and Table 3 provides a descriptive summary of the identified techniques.

**Table 3.** Behavior change techniques in the Mindset smartphone intervention for depression.^a^

Rank	BCT^b^ category	Top module	Pages, n (%)	Mean per module when present (SD)	Most common BCTs used	Intervention, n (%)	Examples of implementation
1	Shaping knowledge	1	205 (63.1)	25.6 (22.5)			
					Instruction on how to perform a behavior	140 (43.1)	Description of how to plan activities (eg, check schedule and anticipate barriers)
					Information about antecedents	65 (20.0)	Changes in season can lead to low mood
2	Repetition and substitution	1	138 (42.5)	17.3 (13.9)			
					Behavioral practice or rehearsal	110 (33.8)	Logging 3 activities per day in app
					Habit formation	16 (4.9)	Repeated practice of activity scheduling or planning
3	Feedback and monitoring	1	113 (34.8)	14.1 (12.4)			
					Self-monitoring of behavior or thought	55 (16.9)	Rate mood after activity
					Feedback on behavior	45 (13.8)	Feedback on cognitive restructuring quiz
4	Self-belief	1	89 (27.4)	11.1 (11.0)			
					Self-talk	41 (12.6)	Textbox to write an encouraging note to self
					Verbal persuasion about capability	38 (11.7)	You're well-equipped to handle future challenges
5	Goals and planning	8	87 (26.8)	10.9 (6.1)			
					Action planning	21 (6.5)	Plan for anticipated stressful events
					Goal setting (behavior)	21 (6.5)	Set intentions for use of Mindset
6	Identity	1	58 (17.8)	7.3 (12.2)			
					Framing or reframing	45 (13.8)	Identify negative beliefs, reframe with a balanced perspective
					Valued self-identity	10 (3.1)	Choose positive qualities about self from the list
7	Comparison of behavior	1	58 (17.8)	7.3 (5.4)			
					Demonstration of behavior	55 (16.9)	Example of negative thinking pattern
					Social comparison	3 (0.9)	Describe another person and select their characteristics from a list
8	Natural consequences	1	51 (15.7)	6.4 (10.8)			
					Information about emotional consequences	35 (10.8)	Negative thoughts may make you feel discouraged
					Information about social or environmental consequences	11 (3.4)	Low mood may lead to social interaction avoidance
9	Associations	1	51 (15.7)	6.4 (5.7)			
					Prompts or cues	51 (15.7)	Encouragement to return to Mindset to refresh skills
10	Comparison of outcomes	1	13 (4.0)	1.6 (4.6)			
					Credible Source	13 (4.0)	Embedded links to evidence-based resources
11	Antecedents	4-6	6 (1.8)	0.8 (1.0)			
					Restructuring the physical environment	3 (0.9)	Enabling "do not disturb" during mindfulness practice
					Body changes	3 (0.9)	Bring attention to breath during mindfulness practice
12	Social support	1	6 (1.8)	0.8 (1.6)			
					Social support (practical)	4 (1.2)	Send messages to ask therapist how to use Mindset
					Social support (emotional)	1 (0.3)	Encouragement to reach out to 911 or 988 in an emergency
13	Reward and threat	8	3 (0.9)	0.4 (1.1)			
					Self-reward	3 (0.9)	Acknowledge how far you've come and skills gained

a Behavior change techniques were coded 878 times throughout all 8 intervention modules; as such, percentages are reported as a percentage of the total app pages.

b BCT: behavior change technique.

### High-Frequency Techniques (≥10%)

Six BCT categories were found in all 8 modules: shaping knowledge (205/325, 63.1%), repetition and substitution (138/325, 42.5%), feedback and monitoring (113/325, 34.8%), self-belief (89/325, 27.4%), comparison of behavior (58/325, 17.8%), and associations (51/878, 15.7%). Goals and planning (87/325 26.8%) and identity (58/325, 17.8%) were present in all modules, except Module 2, where participants began learning to monitor thoughts and activities. Natural consequences (51/325, 15.7%) were present in more than half of the modules.

### Low-Frequency Techniques (<10%)

Comparison of outcomes, social support, antecedents, and reward and threat occurred on fewer than 5% of pages. No techniques were coded from the regulation, scheduled-consequences, or covert-learning categories.

### CBT and BCT Co-Occurrence

**Figure 3.**
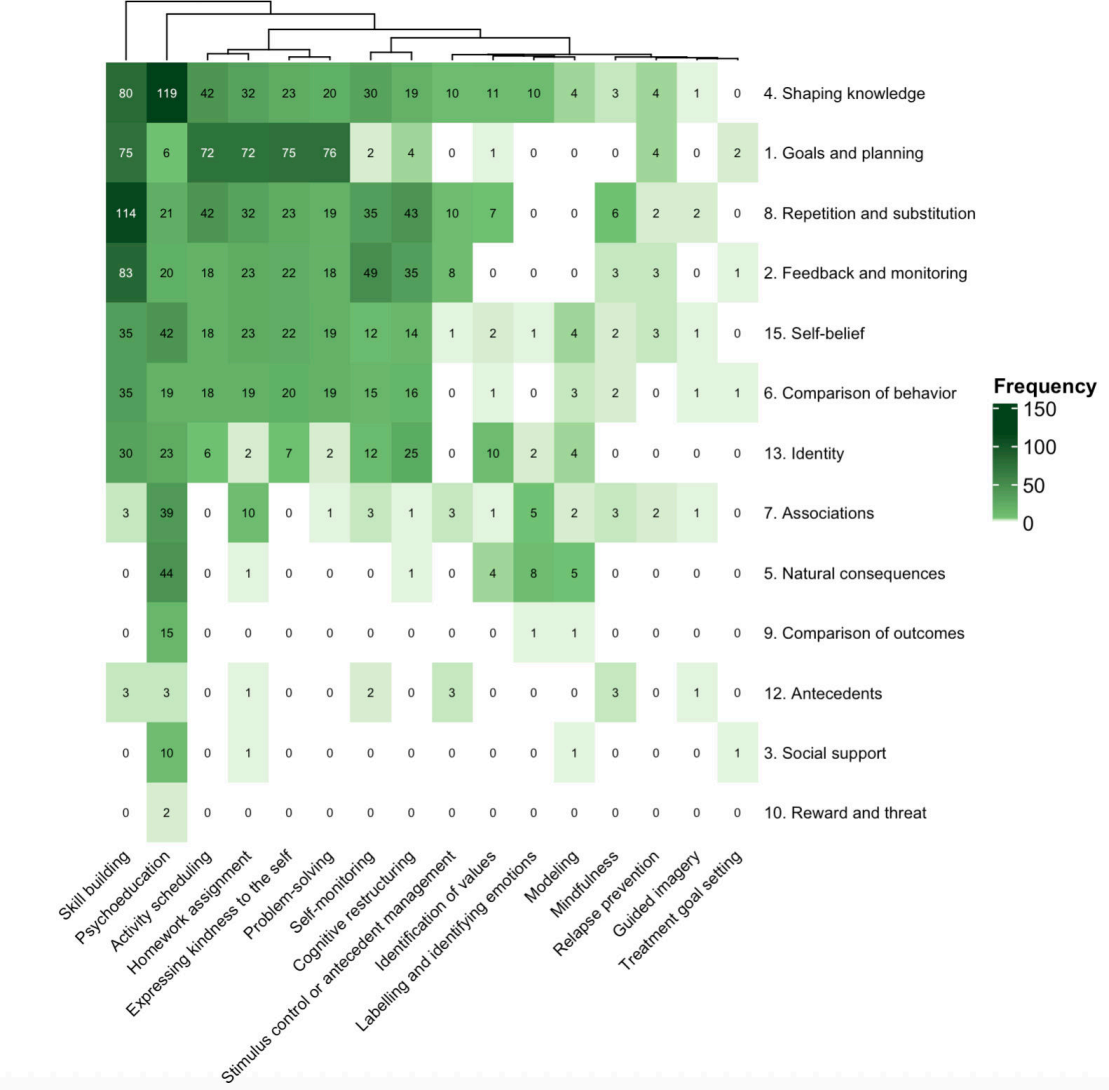
Mapping of cognitive behavioral therapy techniques to behavior change technique categories in the Mindset digital cognitive behavioral therapy intervention for depression. This heatmap displays how often each cognitive behavioral therapy technique (*x*-axis) was coded with each behavior change technique category (*y*-axis) based on page-level content coding of the Mindset smartphone program for adults with major depressive disorder. Cell shading reflects co-occurrence frequency (0‐150; darker cells indicate higher frequency).

We examined how CBT techniques were behaviorally implemented through the codelivery of BCT mechanisms at the page level. Each co-occurrence corresponds to an intervention page where a CBT technique and a BCT are presented simultaneously, aiming to provide insight into how skills are taught, rehearsed, and reinforced during intervention use (Figure 3).

Psychoeducation showed the largest overlap, co-occurring with all 13 observed BCT categories, most frequently with shaping knowledge (119/325, 36.6% of intervention pages had this overlap). Skill building was also widely scaffolded, most often through repetition and substitution (114/325, 35.1%), feedback and monitoring (83/325, 25.5%), and shaping knowledge (80/325, 24.6%). Activity scheduling was consistently behaviorally reinforced, most prominently through goals and planning (72/325, 22.2%), repetition and substitution (42/325, 12.9%), and shaping knowledge (42/325, 12.9%).

Several core CBT techniques were behaviorally supported at moderate densities. For example, cognitive restructuring was frequently paired with repetition and substitution (43/325, 13.2%) and feedback and monitoring (35/325, 10.8%). Self-monitoring and problem solving were supported by fewer BCT categories (generally 2%‐8% of pages per pairing).

By contrast, mindfulness, guided imagery, and relapse prevention were rarely paired with BCTs, with most co-occurrences appearing on ≤10 pages (≤3.1%). Motivational, consequence-based, and social mechanisms, including reward and threat, social support, comparison of outcomes, and antecedents, were similarly infrequent (≤20 pages, ≤6.2% each).

## Discussion

### Principal Findings

This study aimed to advance transparency and mechanistic understanding in digital mental health by conducting a comprehensive analysis of Mindset, a therapist-guided smartphone CBT intervention for depression. Three primary findings emerged. First, all 16 CBT techniques were present across the intervention’s 8 modules, with a total of 528 instances. Second, Mindset incorporated 37 unique BCTs from 13 of 16 taxonomy categories, coded 878 times throughout the intervention. Shaping knowledge, repetition and substitution, and feedback and monitoring were most prevalent. Third, co-occurrence analysis highlighted the behavioral mechanisms through which CBT strategies were operationalized. High-frequency techniques such as psychoeducation, skill building, and activity scheduling were consistently paired with multiple BCTs that structure practice (repetition and substitution), provide feedback (feedback and monitoring), and support learning (shaping knowledge). In contrast, lower-frequency techniques such as mindfulness and guided imagery showed minimal BCT scaffolding, suggesting they were introduced but not behaviorally reinforced through repeated practice. These findings show how Mindset accomplishes fidelity to CBT protocols through multilayered behavioral mechanisms while establishing a replicable methodology for describing therapeutic content in a digital intervention.

### Mechanism Mapping: Showing Implementation Depth Through Behavioral Scaffolding

This study introduces a mechanism mapping approach that directly addresses limitations of prior evaluations of digital CBT by methodologically examining how therapeutic techniques are implemented in user-facing content [25,43,44,63,64]. Specifically, our co-occurrence analysis (Figure 3) builds on previous studies by showing which BCTs scaffold each CBT strategy, making implementation depth visible and quantifiable.

Applying this mechanism mapping approach to Mindset highlighted considerable variation in implementation depth across CBT strategies. High-frequency techniques showed dense scaffolding: psychoeducation drew upon all 13 BCT categories, engaging users through informational content (shaping knowledge), emotional reflection exercises (natural consequences), and repeated practice activities (repetition and substitution). In contrast, mindfulness and guided imagery appeared with minimal BCT support, suggesting in-app introduction without behavioral reinforcement. The content distribution also showed app design choices: Module 1 was the largest (135 pages vs 9‐28 in other modules) and most densely scaffolded, front-loading introductory skills with instruction, practice, and feedback, laying the groundwork for subsequent modules to build upon.

This variation in implementation would be invisible using CBT-only classification but may help explain variability among apps claiming to deliver “CBT-based” care. Evidence from component-focused meta-analyses reinforces why implementation depth matters: combined cognitive and behavioral processes mediate digital intervention outcomes, but techniques delivered on their own showed no effects [45]. Additionally, skill enactment, actively practicing therapeutic strategies, predicts clinical improvement in most trials, whereas knowledge acquisition alone does not [32]. In Mindset, the BCTs supporting behavioral activation and cognitive change, such as instruction, repeated practice, and ongoing monitoring, appeared consistently across modules, creating structured opportunities for skill rehearsal. The intervention was designed this way so that users actively had to practice and rehearse strategies, not just learn about them. Most importantly, mechanism mapping generates testable hypotheses about content features that may facilitate clinical effects. To our knowledge, no other digital mental health study has visualized or described these relationships, providing a novel analytical framework that is replicable across interventions.

### Positioning Mindset Within the Digital Mental Health Landscape

Mindset’s 16 CBT strategies and 37 BCTs exceed typical implementation in digital mental health tools. Prior assessments show that most digital tools include only a median of 3‐4 CBT-based elements [25,29] and 9‐15 BCTs, with conversational agents averaging 15 BCTs (range 4‐30) [63], eating disorder interventions averaging 14 BCTs (range 9‐18) [43], and general mental health apps containing 1‐10 techniques [65]. Our higher technique count compared to prior reviews likely reflects methodological differences in content analysis. We examined every page of the actual intervention, where previous studies coded app store descriptions or published summaries [41,44,63,64], which risks overlooking embedded therapeutic elements and overrelying on developer claims. However, technique quantity alone is not a reliable predictor of treatment effects [65]. Interventions grounded in established psychological theory show better outcomes than those lacking theoretical grounding [43], and full CBT packages outperform single-component strategies. Accordingly, Mindset’s extensive technique involvement may matter less than how these techniques are combined and delivered.

The most frequent CBT techniques in Mindset align with what systematic reviews consistently identify in digital interventions: psychoeducation (present in 50%‐75% of apps), behavioral activation including activity planning and scheduling (31%‐68%), and cognitive restructuring (31%‐79%) [25,27,29]. However, many techniques we identified in Mindset are inconsistently reported in large-scale content syntheses. Skill building, for example, appears in 0%‐12% of interventions depending on how broadly it is defined, with some reviews only capturing specific skill types such as social skills training [25,27]. Similarly, techniques such as homework assignment, self-compassion or expressing self-kindness, problem-solving, stimulus control, and emotion labeling were present in Mindset but are rarely systematically documented in existing app reviews. This inconsistency may reflect the absence of standardized CBT technique taxonomies in digital mental health research, making cross-study comparisons difficult and potentially underestimating the therapeutic content actually delivered to users.

The BCTs most strongly associated with engagement in mobile health interventions, feedback on behavior, self-monitoring, and instructions on how to perform a behavior were among Mindset’s most frequently used [41]. Importantly, these behavioral strategies were not features exclusively designed to keep users engaged; they were directly tied to CBT skill enactment (eg, mood monitoring implemented in service of behavioral activation). This integration contrasts sharply with common commercial designs in which self-monitoring or reminders are detached from core therapeutic mechanisms [25,41]. Mindset’s approach of embedding engagement-related BCTs within therapeutic workflows may explain how the intervention maintains both user engagement and therapeutic fidelity.

Our comprehensive content analysis also sheds light on which CBT strategies received minimal behavioral support and which BCT categories were absent entirely. Techniques such as grounding and mindfulness appeared infrequently and were associated with few BCTs. However, this may reflect the implementation format rather than inadequate support: these strategies were delivered through video demonstrations, which our text-based coding may have undercounted compared to written exercises that explicitly name behavioral techniques. Additionally, entire BCT categories were absent from Mindset, including regulation (eg, reduce negative emotions and conserve mental resources) and scheduled consequences (eg, punishment and reward). However, transparency about what is missing must be interpreted cautiously. Identifying absent strategies or mechanisms does not indicate whether their inclusion would improve outcomes. The value of this analysis lies in enabling transparent evaluation of what tools actually deliver, allowing clinicians and researchers to generate testable hypotheses about which gaps, if any, meaningfully impact outcomes.

### Limitations

This analysis has several limitations. First, the study did not statistically link participants’ exposure to or adherence with these techniques to clinical outcomes, preventing examination of which techniques may have been associated with these outcomes. Second, the review focused on text-based content, potentially underestimating exposure to interactive audio or video elements that may be more engaging than their script counterparts. Third, despite formal BCT training, content coding may have introduced subjective biases. Fourth, Mindset includes therapist-delivered content that was intentionally excluded to isolate digital components, meaning this analysis may underrepresent the full therapeutic exposure users received. Finally, our analysis concentrated on evidence-based CBT techniques and BCTs, without assessing non–evidence-based or potentially contraindicated content. Some mental health apps include elements that may conflict with recommended approaches, which may also impact safety and effectiveness. Accordingly, mechanism mapping should therefore be seen as an initial foundation for objective fidelity monitoring instead of a complete measure of treatment quality.

### Broader Implications

This study demonstrates that transparent evaluation of digital mental health interventions is feasible and necessary. We offer the following actionable recommendations for advancing quality and accountability in the field. For intervention developers, we recommend auditing existing tools using this dual-coding framework to identify which therapeutic techniques lack behavioral scaffolding so that we can confirm techniques are appropriately reinforced through user action. For researchers, we recommend contributing to transparency by reporting the frequency of therapeutic techniques, making this standard practice to enable meta-analyses to examine implementation depth as a moderator of outcomes. Our methodology is fully replicable for any text-based intervention. We encourage clinicians to evaluate apps based on documented content rather than marketing claims, to seek evidence of technique coverage and reinforcement of mechanisms, and transparent reporting of delivered content. This can be used to inform patient-centered recommendations of these tools. Finally, for the field, we hope that this report sets a new precedent for minimum reporting standards when evaluating these tools, so that they can be more comprehensively evaluated and compared.

### Conclusions

The rapid global adoption of digital mental health tools has outpaced our ability to evaluate what they actually deliver. This study introduces mechanism mapping as the first systematic approach to describe which therapeutic strategies are present and how they are behaviorally operationalized through user actions. Unlike prior content analyses that rely on presence or absence coding, our dual-framework methodology reveals implementation depth, distinguishing between techniques that are mentioned versus those supported through repeated practice, feedback, and skill consolidation. This innovation addresses a fundamental transparency gap: most digital interventions function as “black boxes,” making it impossible for clinicians, patients, or researchers to assess therapeutic fidelity.

This replicable framework brings 3 critical contributions to the field: it enables comparative evaluation of digital interventions based on therapeutic content rather than marketing claims, provides testable hypotheses about which implementation features drive clinical effects, and establishes a methodological standard for transparent reporting. In the real world, this work supports clinicians in making evidence-based referrals, empowers patients to choose interventions aligned with their therapeutic needs, and guides developers in creating tools with demonstrable fidelity to evidence-based practices.

Future work should extend this approach by integrating behavioral measurement and mechanistic outcomes, applying mechanism mapping across multiple digital interventions to identify common implementation signatures, exploring whether implementation patterns could inform adaptive tailoring of behavioral supports, and incorporating therapist-delivered components in hybrid models to capture the full therapeutic ecosystem. Importantly, transparency must become the standard in digital mental health, not the exception. Understanding how apps work, for whom, and through what processes is critical for the next generation of precision mental health care.

## Supplementary material

10.2196/84030 Multimedia Appendix 1 Cognitive behavioral therapy and behavior change technique coding descriptive data.

10.2196/84030 Checklist 1 TIDieR framework checklist.

## Abbreviations

BCT: behavior change technique
BCTTv1: behavior change technique Taxonomy version 1
CBT: cognitive behavioral therapy
COM-B: Capability, Opportunity, Motivation–Behavior
HIPAA: Health Insurance Portability and Accountability Act
TIDieR: Template for Intervention Description and Replication
