# Supplementary material for: Identifying Evidence-Based Strategies in a Digital Mental Health Intervention for Depression: Qualitative Content Analysis
Source: J Med Internet Res. 2026 Apr 16;28:e84030. doi: 10.2196/84030 (PMC13086263; doi:10.2196/84030)
Supplement: Checklist 1 [file jmir-v28-e84030-s002.docx]

| **Item** | **TIDieR Requirement** | **How Mindset BCT/CBT study meets requirements** |
| --- | --- | --- |
| **1. Brief name** | Provide the name or a phrase describing the intervention. | The intervention is named Mindset, a therapist-assisted smartphone cognitive-behavioral therapy (CBT) program for depression, consisting of eight modules delivered over eight weeks. |
| **2. Why** | Describe the rationale, theory, or mechanisms of action. | The intervention is grounded in CBT theory, which proposes that changes in thoughts, behaviors, and emotional responses lead to improvements in depression. The paper explains that Mindset aims to modify unhelpful thoughts and increase engagement in meaningful activities through CBT-based skills. |
| **3. What – Procedures** | Describe the procedures, activities, and processes used. | The Methods section details participant-facing procedures: independent app use, completion of module content, logging daily activities and mood in Module 2, scheduling behavioral activation activities from Modules 3–8, and attending brief weekly therapist sessions to review progress and provide support. |
| **4. What – Materials** | Describe the physical or informational materials used. | The paper specifies that the intervention consists of 393 unique app pages (943 including repeats), delivered primarily via in-app text with some pages (introductory content and mindfulness/grounding exercises) also provided via embedded video. All therapeutic materials are accessible exclusively within the Mindset smartphone app. Table 1 summarizes module content. |
| **5. Who provided** | Describe the intervention providers, including expertise/training. | Weekly support was provided by licensed clinical psychologists trained in CBT for depression. Their role was to review progress, troubleshoot barriers, and support engagement while participants used the app. |
| **6. How** | Describe the modes of delivery. | Mindset was delivered as a self-guided smartphone app, supplemented by remote weekly therapist sessions conducted via secure telehealth or phone. Participants interacted with the app individually on their own devices. |
| **7. Where** | Describe the types of locations where the intervention occurred. | Because therapists were licensed in Massachusetts, participants were required to reside in Massachusetts. Mindset was used on participants’ own smartphones in their everyday environments (e.g., home and community settings), while weekly therapist sessions were delivered remotely via secure telehealth or phone. |
| **8. When and how much** | Describe the number of sessions, schedule, duration, and intensity. | The full intervention was delivered over 8 weeks, with one module released per week. Module 1 could be completed at the participant’s own pace; Modules 2–8 required a minimum of 7 days before advancement. Participants were instructed to schedule at least three behavioral activation activities per week from Modules 3–8 and to log mood ratings daily when prompted. Weekly therapist sessions lasted approximately 15–20 minutes. Participants could revisit completed content at any time. |
| **9. Tailoring** | Describe any personalization, titration, or adaptation. | Tailoring occurred through participant-selected activities: from Module 3 onward, participants chose behavioral activation tasks from a bank of 102 activities organized into seven core values (e.g., health and wellness, relationships, creative pursuits) and scheduled the ones most meaningful to them. |
| **10. Modifications** | Describe any changes made during the study. | The paper notes that no modifications were made to the Mindset intervention during the trial or coding process. The only adjustment was an analytic weighting of repeated scheduling pages to prevent overestimation of exposure; the intervention content itself remained unchanged. |
| **11. Planned fidelity assessment** | Describe how adherence/fidelity was planned to be assessed. | Fidelity in this study refers to coding fidelity, not participant adherence. The Coding Procedures section describes a planned process: independent initial coding by the first author, review and memoing by two additional coders, use of formal CBT and BCTTv1 definitions to guide decisions, and consensus discussions [3, 4]. Interrater agreement was quantified (percentage agreement) prior to consensus. These procedures served as the planned fidelity monitoring approach for content coding. |
| **12. Actual fidelity / adherence** | Describe the extent to which the intervention was delivered as planned. | The paper states that the analysis focused on embedded therapeutic content and did not measure individual participants’ behavioral adherence or dosage (e.g., actual frequency of mindfulness practice or completed activities). This limitation is acknowledged explicitly in the Limitations section, noting that the degree to which specific techniques influenced outcomes remains unknown. |

Multimedia Appendix 2 – Alignment with TIDieR framework [1]

**References**

1. Hoffmann, T.C., et al., *Better reporting of interventions: template for intervention description and replication (TIDieR) checklist and guide.* Bmj, 2014. 348.
2. Wilhelm, S., et al., *Feasibility, Acceptability, and Preliminary Efficacy of a Smartphone App–Led Cognitive Behavioral Therapy for Depression Under Therapist Supervision: Open Trial.* JMIR Mental Health, 2024. 11(1): p. e53998.
3. Michie, S., et al., *The behavior change technique taxonomy (v1) of 93 hierarchically clustered techniques: building an international consensus for the reporting of behavior change interventions.* Annals of behavioral medicine, 2013. 46(1): p. 81-95.
4. Wasil, A.R., et al., *A review of popular smartphone apps for depression and anxiety: assessing the inclusion of evidence-based content.* Behaviour research and therapy, 2019. 123: p. 103498.
